# A New SteatoScore in the Evaluation of Non-Alcoholic Liver Disease in Oncologic Patients

**DOI:** 10.3389/fonc.2022.873524

**Published:** 2022-04-27

**Authors:** Dania Cioni, Michela Gabelloni, Andrea Sanguinetti, Laura De Rosa, Giacomo Aringhieri, Rachele Tintori, Gianvito Candita, Maria Febi, Francesco Faita, Riccardo Lencioni, Emanuele Neri

**Affiliations:** ^1^Department of Surgical, Medical, Molecular Pathology and Emergency Medicine, University of Pisa, Pisa, Italy; ^2^Academic Radiology, Department of Translational Research, University of Pisa, Pisa, Italy; ^3^Hepatology Unit, Pisa University Hospital, Pisa, Italy; ^4^Institute of Clinical Physiology, National Research Council, Pisa, Italy

**Keywords:** SteatoScore, non-alcoholic fatty liver disease, ultrasound, computed tomography, oncology

## Abstract

**Purpose:**

The aims of this study were to evaluate the reproducibility of a new multi-parametric steatoscore (new SteatoScore) in oncologic patients with non-alcoholic fatty liver disease (NAFLD) and to compare it with computed tomography (CT).

**Materials and Methods:**

Fifty-one (31 men, 20 women) oncologic patients, with a mean age and weight of 63.9 years and 78.33 kg, respectively, were retrospectively enrolled in the study. Patients underwent ultrasound (US) and computed tomography (CT) examinations as part of their oncologic follow-up protocol. US examinations were performed by using a 3.5-MHz convex probe. During the US examination, three standardized clips were obtained in each patient. Two operators performed all measurements, one of whom repeated the processing twice in 1 year. Hepatic/renal ratio (HR), attenuation rate (AR), diaphragm visualization (DV), hepatic/portal vein ratio (HPV), and portal vein wall visualization (PVW) were acquired and calculated by using Matlab and inserted in a multi-parametric algorithm called new SteatoScore. On unenhanced CT scan, hepatic attenuation (HA), liver-spleen difference (L-S), and liver/spleen ratio (L/S) were measured by placement of a region of interest (ROI) within liver and spleen parenchyma, avoiding areas with vessels and biliary ducts.

**Results:**

The intra-observer variability was greater than the inter-observer one, with intraclass correlation coefficient (ICC) values of 0.94 and 0.97, respectively. Correlation between single US and CT parameters provided an agreement in no case exceeding 50%. New SteatoScore showed high reproducibility, and high coefficient of correlation with L-S (*R* = −0.64; *p* < 0.0001) and L/S (*R* = −0.62; *p* < 0.0001) at CT.

**Conclusion:**

New SteatoScore has a high reproducibility and shows a good correlation with unenhanced CT in evaluation of oncologic patients with NAFLD.

## Introduction

Non-alcoholic fatty liver disease (NAFLD) has become a major public health challenge and a global epidemic. NAFLD is the most common liver disorder in Western countries, affecting 17%–46% of adults (1). NAFLD is often asymptomatic and is considered an indolent liver pathology unless it is complicated by inflammation, leading to non-alcoholic steatohepatitis (NASH), which may progress to fibrosis, cirrhosis, and hepatocellular carcinoma (2). NAFLD is associated with metabolic syndrome (3, 4), increased mortality from cardiovascular disease (4), and chronic kidney disease (5). Chemotherapeutic agents may also lead to NAFLD (6). 5-fluorouracil (5-FU) and irinotecan, chemotherapeutic agents administered to patients with colorectal cancer with metastases, have been reported to be closely related with chemotherapy-induced fatty liver disease (7, 8).

Hepatic steatosis is characterized by excessive triglyceride accumulation and deposition of lipid vesicles within the cytoplasm of hepatocytes; either microvesicular or macrovesicular steatosis can be observed in hepatic steatosis. The overlap of lobular inflammation and ballooning degeneration of hepatocytes and eventually of fibrosis are suggestive of steatohepatitis (9). Concerning pathogenesis, chemotherapeutic agents promote oxidative stress and mitochondrial accumulation of large amounts of reactive oxygen species not only in cancer cells but also in normal hepatocytes, inducing the deposition of lipid vesicles, which can be diffuse or focal (6–9).

Because of the important clinical impact of this disease, diagnosis and quantification of hepatic fatty infiltration are fundamental. The gold standard for assessment of fatty liver is liver biopsy (10), but it is an invasive method. Proton magnetic resonance spectroscopy (^1^H-MRS) has shown to provide a sensitive, accurate, and quantitative evaluation of liver fat content (11). Although the correlation between liver biopsy and ^1^H-MRS measurements has already been reported (11, 12), the use of this approach remains limited because of the high costs and low availability of magnetic resonance equipment.

In this context, noninvasive assessment of NAFLD has a great importance: an easy-to-use tool allowing simple noninvasive evaluation of liver fat content in different clinical settings would be beneficial for identification of asymptomatic high-risk patients, evaluation of appropriate therapy response, and detecting disease progression in diagnosed patients.

The so-called SteatoScore is an ultrasound (US)-based system for the non-invasive assessment of liver fat content that has been validated using MR as the gold standard. The algorithm quantifies fat accumulation in the liver by combining five different US parameters to provide a single index, the SteatoScore, which is representative of intra-hepatic fat content and can be used in clinical practice to discriminate between the presence and absence of steatosis. With respect to diagnostic performance, the ability of SteatoScore to discriminate between the presence and the absence of steatosis was confirmed by the results obtained with the ROC curve, namely, a high AUROC value as well as specificity and sensitivity of approximately 90% (13).

In the last year, a new SteatoScore algorithm has been developed using a larger group of patients who underwent both MR and US. This new score, called new SteatoScore, combines four of the five original parameters.

Other than the SteatoScore, there are previous different US scores with the aim to quantify steatosis, like the US-fatty liver indicator (FLI) (14) and the Hamaguchi score (15). However, these scores provide a qualitative analysis of the US image and then they try to give a quantitative number of the steatosis. Otherwise, the SteatoScore does not require a physician’s opinion on the clips acquired. Similar to SteatoScore, a simpler score was created in 2009, the so-called Hepatorenal steatosis index (16), considering exclusively the association between the echogenicity of the liver and the kidney.

There is a constant relationship between the mean CT attenuation of the liver and spleen in normal individuals (17). For this reason, over the years, various criteria for diagnosing steatosis using unenhanced CT have been proposed. The most used parameters reported in literature are hepatic attenuation (HA), liver-to-spleen ratio (L/S), and liver-to-spleen difference (L-S). Kodama et al. (18) found that hepatic attenuation of 40 HU represents fatty change of approximately 30%. Park et al. (19) performed a study comparing HA, L/S, and L-S to determine the presence of steatosis equal to or greater than 30%. They found that cutoff values of 0.9 and 58 HU for L/S and HA, respectively, provided high values of both sensitivity and specificity. However, CT is not an accurate method to diagnose hepatic steatosis, because it shows a limited accuracy for detecting mild degree hepatic steatosis (19). Also, the presence of excessive iron (hemochromatosis, hemosiderosis) and ingestion of several drugs including amiodarone (which contains iodine and accumulates in the liver) increase the attenuation value of liver parenchyma on unenhanced CT scan (20–22).

The purpose of this study was to evaluate the reproducibility of the new SteatoScore in a series of oncologic patients with NAFLD and to compare it with CT. The group of oncologic patients had to perform a CT scan periodically as part of their follow up. In this group of patients, the degree of hepatic steatosis (using HA, L/S, and L-S) at CT scan was measured and compared with the new SteatoScore.

## Materials and Methods

Fifty-one consecutive oncologic patients were enrolled in the study between January 2019 and September 2019 in our center (Academic Radiology, University of Pisa). Informed consent for data collection was obtained from each patient. There were 31 male and 20 female patients. The mean age was 63.92 ± SD 12.44 years old, mean weight was 78.33 ± SD 16.71 kg, mean height was 168.9 ± SD 8.21 cm, and mean BMI was 27.43 ± SD 5.4. No patient had a history of alcoholic abuse. Of 51 patients with NAFDL, 8 (15.7%) had diabetes and 12 (23.5%) had hyperlipidemia. In 13 patients (25.5%), weight loss was observed after surgery (ranging from −4 kg to −13 kg). All patients received chemotherapy before ultrasound examination and only 7 patients (13.7%) were receiving hormonotherapy at the time of the examination. Patients underwent US examination as part of their oncologic follow-up protocol. Among 51 patients, 19 patients had been previously treated for gastrointestinal cancer, 9 for breast cancer, 6 for kidney cancer, 5 for lung cancer, 3 for neuroendocrine pancreatic cancer, 3 for ovarian cancer, 2 for prostate cancer, 2 for testicular cancer, 1 for thyroid cancer, and 1 for adrenal gland cancer. Of 51 patients, 35 were not treated with chemotherapy at the time of US examination. Nine of the patients had documented liver storage diseases, chronic hepatitis, and cirrhosis. All patients had to have at least an unenhanced CT scan of the abdomen performed within the previous year as part of their oncologic follow-up protocol.

### CT Examination

An unenhanced CT scan of the abdomen as part of an unenhanced and contrast-enhanced CT study of all patients was carried out on multivendor 64-row CT scanners (Discovery CT750 HD or LightSpeed VCT, General Electric, Milwaukee, WI; and Siemens Somatom Sensation, Siemens Healthineers). CT protocol included the acquisition of an unenhanced scan of the abdomen and the acquisition of arterial, venous, and delayed phases of the lung and abdomen after bolus injection of 100–150 ml of contrast media (Iomeron 400, Bracco, Milan, Italy) with an injection rate of 4 ml/s, followed by bolus saline injection of 40–50 ml with an injection rate of 4 ml/s. Arterial phase of the abdomen was acquired 35 s after the start of injection, venous phase (including the lungs) after 70 s, and delayed phase of the abdomen after 100 s. While three different multidetector CT scanners were used for the acquisition of CT images, the scan and contrast medium protocols used were similar and standardized, leading to comparable and consistent image quality across all patients. In particular, the use of a tube voltage of 120 kV in all patients contributed to obtain consistent CT attenuation numbers on both unenhanced and contrast-enhanced images.

Unenhanced and contrast-enhanced images were acquired with a thickness of 2.5–3 mm and reconstructed at 1.25- to 1.5-mm slice intervals using soft tissue reconstruction algorithms. According to Park et al. (19) and Kodama et al. (18), hepatic attenuation (HA), liver-spleen difference (L-S), and liver/spleen ratio (L/S) were measured by the placement of a region of interest (ROI) on unenhanced images within right hepatic lobe and spleen parenchyma avoiding areas with vessels and biliary ducts.

### Quantitative US Examination

**Figure 1:**
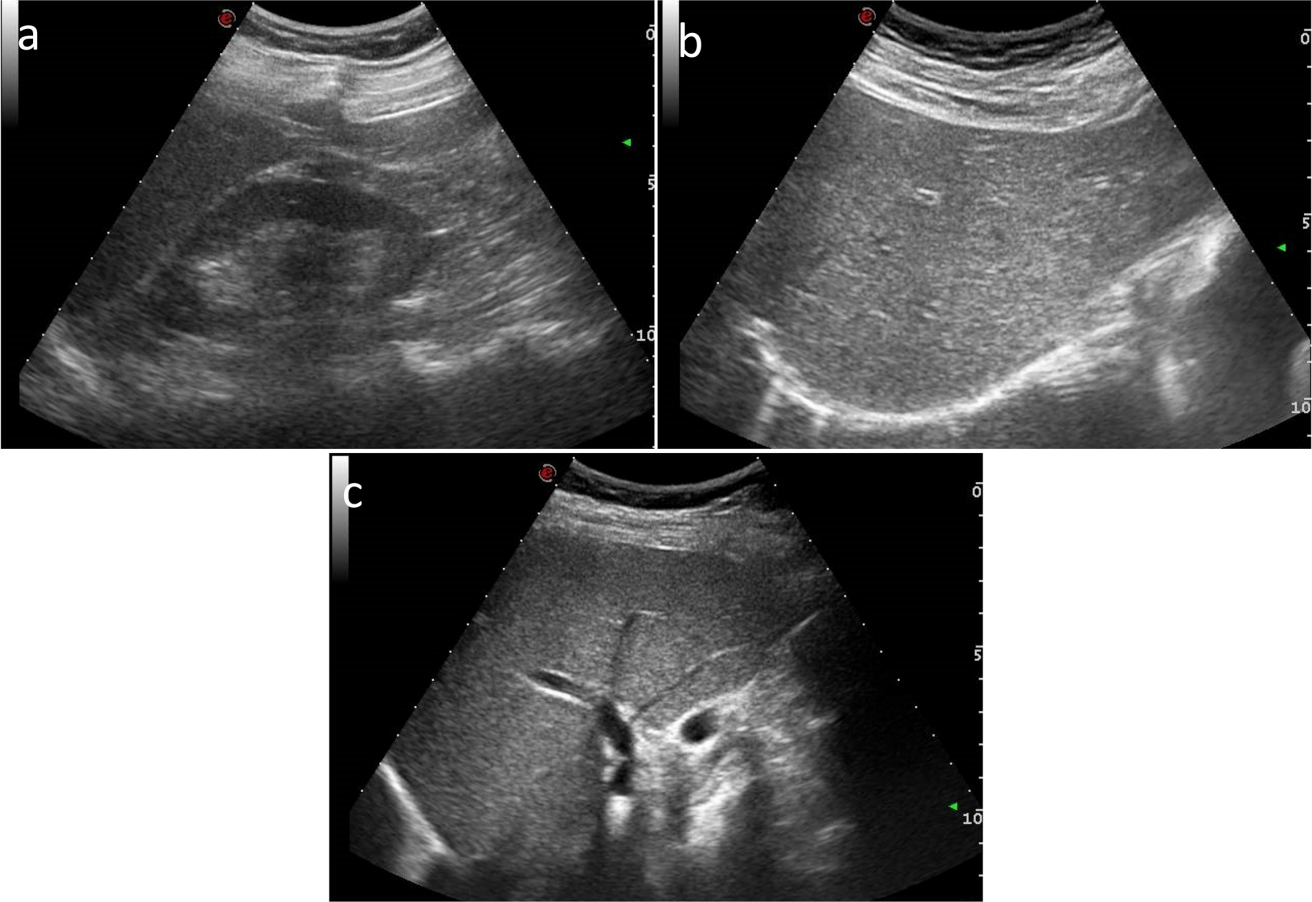
US subcostal longitudinal scan images of **(A)** both liver and right kidney, **(B)** liver and right diaphragm, and **(C)** the wall of intrahepatic portal vein.

US examinations were performed by using US equipment (MyLab™Twice, Esaote, Genova, Italy) in association with a 3.5-MHz convex probe. After a conventional US examination of the abdomen, three standardized clips were obtained in each patient: (a) a longitudinal subcostal scan so that both liver and right kidney were clearly visualized on the same level (**Figure 1A**); (b) a longitudinal subcostal scan to clearly visualize liver parenchyma and right diaphragm (the left side of diaphragm was included when possible) (**Figure 1B**); and (c) a subcostal longitudinal scan to correctly visualize the wall of intrahepatic wall portal vein (**Figure 1C**). US images were acquired with all filters disabled and scan settings (e.g., gain and image depth) tailored to optimize image quality in every single patient, so as to obtain a homogeneous image quality.

**Figure 2:**
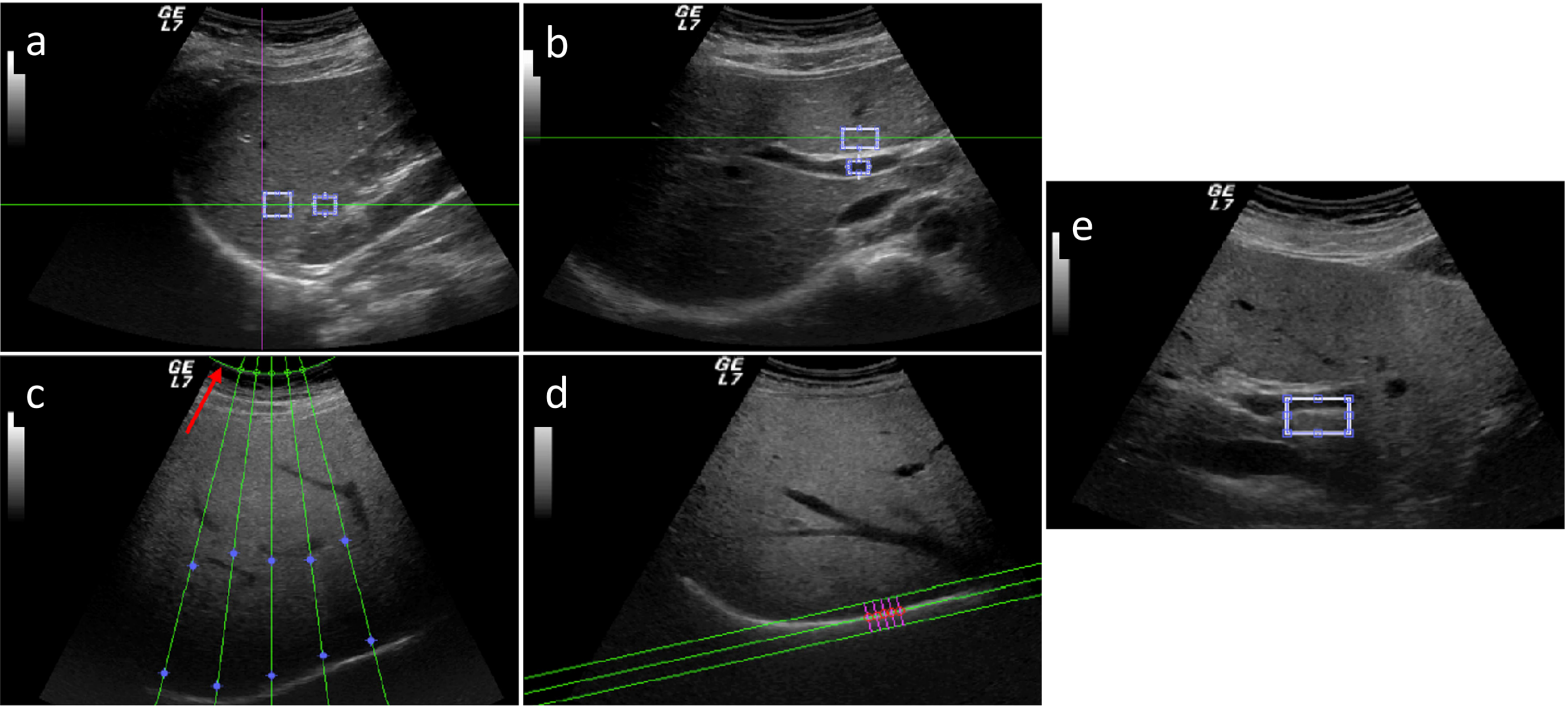
Five parameters obtained by semi-automatically processed US images: hepatic–renal ratio (HR) **(A)**, hepatic portal vein (HPV) ratio **(B)**, attenuation rate (AR) **(C)**, diaphragm visualization (DV) **(D)**, and portal vein wall (PVW) **(E)**.

Five parameters were obtained by semi-automatically processing US images. Hepatic–renal ratio (HR) was based on the analysis of pixel gray levels from two rectangular ROIs, the first one placed in liver parenchyma, avoiding focal hypo- and hyper-echogenicity, and the second one in a corresponding portion of renal cortex without large vessels. HR ratio values were automatically obtained for each frame by dividing the mean gray level of the hepatic ROI by that obtained from renal ROI and averaging over each US clip (**Figure 2A**).

Hepatic portal vein (HPV) was based on the analysis of pixel gray levels from two ROIs, the first one placed in the liver parenchyma, avoiding focal hypo- and hyper-echogenicity, and the second one within the portal vein. HPV ratio values were automatically obtained for each frame by dividing the mean gray level of the hepatic ROI by that obtained from the inner of the portal vein (**Figure 2B**). For measuring attenuation rate (AR), five US profiles within the US beam and corresponding to the liver parenchyma were selected and the attenuation constant was obtained for each profile using a fitted model with a single decreasing exponential curve. AR was calculated as the mean of the five attenuation constants (**Figure 2C**). Diaphragm visualization (DV) was computed by selecting perpendicular profiles in the flattest portion of the diaphragm and the parameter was evaluated as the peak of the mean profile normalized for both the overall gain and depth at which the diaphragm line was located (**Figure 2D**). Portal vein wall (PVW) parameter was calculated from the same US images used for HPV ratio. Two ROIs were manually drawn: the first was placed in the liver parenchyma, avoiding focal hypo- and hyper- echogenicity, and the second was positioned in a corresponding part of the portal vein wall. For each frame, the mean gray level obtained for the liver ROI was normalized to that for the PVW ROI; the final PVW visualization value was calculated as the average of the results obtained from three frames of the clip (**Figure 2E**).

The first clip was used to calculate the hepatic–renal ratio (HR), the second one was used to calculate attenuation rate (AR) and diaphragm visualization (DV), and the third one was used to calculate hepatic/portal vein ratio (HPV) and portal vein wall visualization (PVW).

US clips of each patient were loaded on a custom-made software tool created using Matlab R2020a (Mathworks, Natick, MA, USA) to extract each parameter. For each parameter, we used a specific software tool of Matlab R2020a (for example, the HR parameter was obtained after loading the clip into the software and placing two ROIs within liver parenchyma and kidney parenchyma). This procedure was the same for each parameter extraction. Only ROI placement changed according to the different types of patients and their corresponding images.

The measurement of the five parameters was done by the same operator twice (M1 and M2), with 1 year between the two measurements. During this time, the operator gained more experience, so the comparison between the two assessments is to be considered as a learning curve for the single operator. A third measurement was done by a second operator (L) at the time of M2 measurement. During this time, the first and the second operator followed a more methodical processing protocol, deciding to use the same videoclip’s frames.

### SteatoScore Model

All the previous five US parameters were measured, to assess the SteatoScore mathematical algorithms, which resulted from more years of research and a greater number of patients. This new SteatoScore is based on four of the five US parameters computed on US images, excluding the worst parameter in terms of correlation, and it is expressed by the following equation:


$$\text{Steatoscore }{\text{(\%fat)=10}}^{\left(0.4068\;+\;0.2446\;\times \text{ HR }+\;5.0262\;\times \text{ AR }-\;1.819\;\times \text{ DV }-\;0.0803\;\times \text{ PVW}\right)}$$

### Variability Analysis

To evaluate the variability in the assessment of US parameters and SteatoScore, the following analysis was performed: (a) the intra-observer variability was evaluated between M1 and M2 measurements and a learning curve for the first operator was considered. M1 and M2 measurements were acquired 1 year apart; (b) the inter-observer variability was calculated between M2 and L. All parameters were analyzed both separately and as the multiparametric score (SteatoScore). Intraclass correlation coefficient (ICC) and Bland–Altman analysis were used for the analysis.

### Agreement Between SteatoScore and CT

To evaluate the agreement between US and CT scan, the multiparametric score obtained by the M2 operator was chosen to consider the effects of a proactive training period in the comparison.

The correlation between CT scan parameters (HA, LS ratio, and LS difference) and the SteatoScore was performed comparing the following: (a) single US parameters taken by M2 (HR, AR, DV, HPV, and PVW) and all the CT parameters (HA, L/S, and L-S); (b) the SteatoScore computed by M2 and CT parameters (HA, L/S, and L-S); and (c) the SteatoScore computed by M2 and the percentage of fat as computed using Kodama conversion of HA.

## Results

Among 51 patients, four of them were excluded because the quality of US acquisitions was poor for the assessment of the new SteatoScore. US images were not optimal for the presence of artifacts due to abdominal movements and bowel air. No significant difference was observed in case of overweight and obese patients, i.e., those with BMI values >25 kg/m^2^.

### Results of Intra-Operator (Learning Curve) and Inter-Operator Analysis

The ICC values, the limits of agreement, and the bias assessment between the single US parameters and the SteatoScore computed by operator 1 (intra-operator variability: M1 vs. M2) and between the two operators (inter-operator variability: M2 vs. L) are reported in **Table 1**.

**Table 1:** ICC values, the limits of agreement, and bias assessment between the single US parameters and the SteatoScore computed by operator 1 (intra-operator variability: M1 vs. M2) and between the two operators (inter-operator variability: M2 vs. L).

	ICC		Limits of agreement		Bias	
	M1 vs. M2	M2 vs. L	M1 vs. M2	M2 vs. L	M1 vs. M2	M2 vs. L
AR	0.88	0.94	−0.018 0.01	−0.008 0.013	−0.004	0.003
HR	0.81	0.79	−0.44 0.38	−0.48 0.32	−0.034	−0.081
DV	0.98	0.99	−0.04 0.04	−0.02 0.02	−0.0002	−0.00009
HPV	0.75	0.80	−0.69 1.10	−0.31 0.23	0.020	0.044
PVW	0.85	0.84	−0.53 0.67	−0.45 0.71	0.69	0.13
Steatoscore	0.94	0.97	−0.56 0.33	−0.42 0.26	−0.113	−0.078

All ICC values showed a good reliability, higher than 0.75 with the SteatoScore, showing excellent ICC values of 0.94 and 0.97 for comparison of M1 vs. M2 and M2 vs. L measurements, respectively.

**Figure 3:**
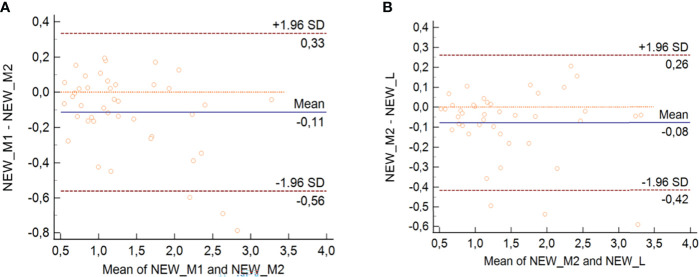
Bland–Altman analysis for intra-operator 1 **(A)** and inter-operator **(B)** with SteatoScore values.

The results for the Bland–Altman analysis of the multiparametric SteatoScore are shown in **Figure 3** for both intra- and inter-operator analysis with the bias and the interval of agreement that were both higher for M1 vs. M2 with respect to M2 vs. L.

### Results of the Agreement Between SteatoScore and CT

The results of the agreement between the US and CT parameters are shown in **Table 2**.

**Table 2:** Correlation between US parameters and SteatoScore and CT parameters (single and with HA Kodama conversion).

	HA (R, p)	L/S (R, p)	L-S (R, p)	CT fat% Kodama
AR	−0.41 <0.01	−0.37 =0.01	−0.44 <0.01	N.A.
HR	−0.32 <0.05	−0.37 <0.05	−0.38 <0.01	N.A.
DV	0.48 <0.001	0.46 =0.001	0.49 <0.001	N.A.
HPV	0.19 =0.22 (n.s.)	0.15 =0.34 (n.s)	0.18 =0.25 (n.s.)	N.A.
PVW	0.25 =0.11 (n.s.)	0.32 <0.05	0.30 =0.05	N.A.
Steatoscore	−0.57 <0.001	−0.62 <0.001	−0.64 <0.001	−0.58 <0.001

n.s., non significant; N/A, not applicable.

The correlations between single US parameters and CT-derived ones were in the absolute range of 0.30–0.49. On the other hand, the new SteatoScore showed a better correlation with all three CT parameters, with a minimum value of *R* = −0.57 and a maximum value of *R* = −0.64.

The Kodama conversion of the CT attenuation parameter (HA) slightly improved its correlation with the SteatoScore from *R* = −0.57 to *R* = −0.58 (**Table 2**).

## Discussion

A new US-based system for non-invasive assessment of liver fat content was developed and tested. The proposed algorithm quantifies fat accumulation in the liver by combining four different US parameters to provide a single index, the new SteatoScore, which is representative of intrahepatic fat content and can be used in clinical practice to discriminate between the presence and absence of steatosis.

Quantification of hepatic fat accumulation is fundamental to obtain information on overall health status, as NAFLD includes different liver pathologic states such as steatosis up to cirrhosis and potentially hepatocellular carcinoma, and may be associated with metabolic syndrome, increased mortality from cardiovascular disease, and chronic kidney disease (2–5). In oncologic patients, moreover, quantification of liver fat accumulation is particularly important, as far as some chemotherapeutic agents may lead to NAFLD (6, 7, 23, 24).

Quantitative US analysis could represent a valid alternative to liver biopsy and ^1^H-MRS, as it is non-invasive, widely available, and relatively inexpensive with respect to liver biopsy and ^1^H-MRS, which are invasive and associated with high cost and low availability (25, 26).

Increased echogenicity of the liver parenchyma due to increased liver fat content can impair the assessment of deep liver structures as a result of lower US beam depth penetration. To overcome this limitation, four US parameters were selected to obtain a quantitative measurement by using standardized clips. According to the results of the study, the new SteatoScore showed better results for inter-reproducibility than for intra-operator analysis. All ICC values were greater than 0.75 with the SteatoScore showing excellent ICC values of 0.97 and 0.94 for comparison of M2 vs. L and M1 vs. M2 measurements, respectively. This can be explained considering that the first operator gained experience over time, and the second operator followed the same processing protocol as the first operator. Therefore, experience and training in calculating SteatoScore parameters is a crucial point, but it does not require a physician experienced in abdominal ultrasound. Bland–Altman analysis of the SteatoScore also showed for both inter-operator and inter-operator analysis with the bias and the interval of agreement higher values for M2 vs. L than for M1 vs. M2. With respect to results of the agreement between the US and CT parameters, correlations between single US parameters and CT-derived ones were in the range of 0.30–0.49. On the other hand, the new SteatoScore showed a high coefficient of correlation with L-S (*R* = −0.64; *p* < 0.0001) and L/S (*R* = −0.62; *p* < 0.0001) at CT.

The study has some limitations. First, CT rather than histology or ^1^H-MRS was used as the imaging technique to compare measurements obtained with the new SteatoScore model. However, liver biopsy is invasive and unsuitable for asymptomatic individuals. Moreover, it provides only small liver specimens, and intrahepatic fat distribution may be inhomogeneous, thus inducing sampling errors. ^1^H-MRS, whose data correlate well with hepatic fat deposition according to previous reports (26), was not available. Promising results have been reported by some authors (27) about the application of the new techniques of artificial intelligence (AI). In fact, as radiomics can analyze the average situation of a whole organ or tissue, it is appropriate for radiomics to evaluate the range and severity of diffuse liver steatosis, thus providing the possibility of non-invasive classification of NAFLD (28–30). Moreover, radiomics can be applied not only to conventional imaging examinations such as CT and MRI, but also to those specific tests in the liver disease field, e.g., elastography (which may have the potential to improve the accuracy of the diagnosis of liver fibrosis) (31). Finally, another limitation of the study is represented by the small number of patients. However, the new SteatoScore showed a high reproducibility and a good correlation with CT in evaluation of patients with NAFLD. As far as oncologic patients may develop NAFLD as a complication of their chemotherapy agents, the new SteatoScore represents a reliable, noninvasive, and low-cost tool that may be useful to demonstrate the presence of liver steatosis. Further investigations are strongly recommended to confirm results of this study and to stratify different grades of liver steatosis by using the new SteatoScore.

## Data Availability Statement

The raw data supporting the conclusions of this article will be made available by the authors, without undue reservation.

## Ethics Statement

Ethical review and approval were not required for the study on human participants in accordance with the local legislation and institutional requirements. The patients/participants provided their written informed consent to participate in this study.

## Author Contributions

DC, FF, RL, and EN: conceptualization. MF, AS, GC, RT, and LR: methodology. MF, AS, GC, and RT: writing—original draft preparation. MG, GA, FF, LR, and DC: writing—review and editing. DC, FF, EN, and RL: supervision and guarantee of scientific integrity. All authors contributed to the article and approved the submitted version.

## Conflict of Interest

The authors declare that the research was conducted in the absence of any commercial or financial relationships that could be construed as a potential conflict of interest.

## Publisher’s Note

All claims expressed in this article are solely those of the authors and do not necessarily represent those of their affiliated organizations, or those of the publisher, the editors and the reviewers. Any product that may be evaluated in this article, or claim that may be made by its manufacturer, is not guaranteed or endorsed by the publisher.
